# Ischemic Colitis Caused by Intra-Aortic Balloon Pump Counterpulsation

**DOI:** 10.1155/2015/747989

**Published:** 2015-11-01

**Authors:** H. El-Halawany, A. Bajwa, M. Shobassy, A. Qureini, R. Chhabra

**Affiliations:** ^1^Division of Gastroenterology and Hepatology, Saint Luke's Hospital of Kansas City, University of Missouri-Kansas City School of Medicine, Kansas City, MO 64108, USA; ^2^Internal Medicine, UMKC School of Medicine, Kansas City, MO 64108, USA; ^3^Section of Gastroenterology/Hepatology, Temple University Hospital, 3401 N. Broad Street, Philadelphia, PA 19140, USA; ^4^Saba University School of Medicine, Church Street The Bottom, P.O. Box 1000, Saba, Dutch Caribbean, Netherlands

## Abstract

Intra-aortic balloon pump counterpulsation (IABP) has been shown to prolong life in critically ill cardiac patients. However, complications including distal emboli, balloon rupture, bleeding, limb loss, and bowel ischemia continue to be associated with them. We present a case of a 56-year-old male who suffered bowel ischemia as a result of a malpositioned IABP. While the benefit of such devices in critically ill patients is not disputed, patients as well as clinicians should be aware of the potential side effects and patients undergoing IABP placement should be monitored for complications.

## 1. Case Report

**Figure 1:**
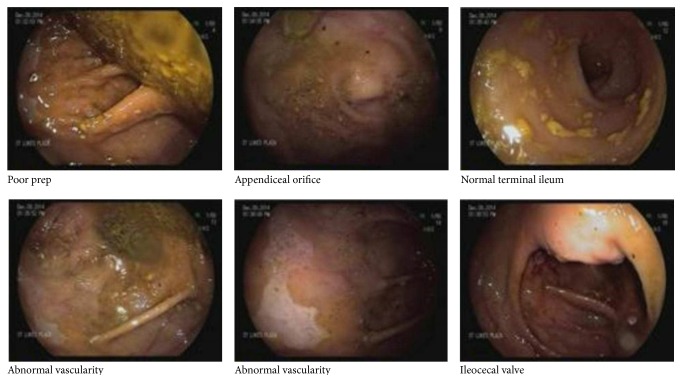
Endoscopic findings of abnormal vascularity, congestion, erosions, nodularity, granularity, erythema, and ulceration of the cecum and ascending colon consistent with ischemic colitis.

**Figure 2:**
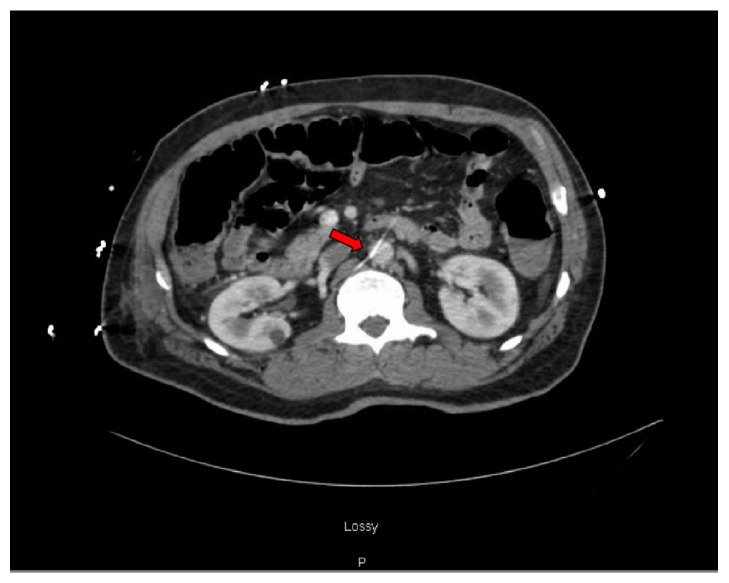
CT abdomen/pelvis findings showing the inferior metallic marker of the intra-aortic balloon pump below the origin of the superior mesenteric artery (arrow).

An African American male in his 50s presented with increased shortness of breath, dyspnea on exertion, and orthopnea. He has a past medical history of coronary artery disease with multiple myocardial infarctions and two coronary stents placed in 2003 and two additional coronary stents placed two months prior to his admission. He had a history of systolic heart failure with a left ventricle ejection fraction of 20%. In the emergency department atrial fibrillation with rapid ventricular rate was noted with a heart rate of 140. A diltiazem infusion was started and he was admitted to the Cardiac Intensive Care Unit. Repeat echocardiogram several days later showed progressive worsening of his ischemic cardiomyopathy with an ejection fraction of 18%. He was started on inotropic support and underwent placement of an intra-aortic balloon pump (IABP) to assist with hemodynamic stability. Approximately 24 hours later the patient had an episode of melena associated with a drop in his hemoglobin from 10.1 to 7.7. He was started on proton pump inhibitor infusion. He underwent emergent esophagogastroduodenoscopy which showed erythema and erosions in the gastric fundus compatible with gastritis as well as grade 1 reflux esophagitis but no evidence of active bleeding. A* Helicobacter pylori *stool antigen was found to be negative. A colonoscopy was performed after hemodynamic stabilization for further evaluation of melena and pretransplant screen. This was positive for abnormal vascularity, congestion, nodularity, erosions, erythema, and ulceration in the cecum and ascending colon suggestive of ischemic colitis which was later confirmed by biopsy (Figure 1). Given the unusual involvement of nonwatershed areas such as the cecum and ascending colon, a CT of the abdomen and pelvis with contrast was performed to evaluate the celiac and mesenteric vasculature. There was no significant narrowing of either vessel noted on the CT; however the inferior metallic marker of the IABP was found to be inferior to the origin of the superior mesenteric artery (Figure 2). Findings were consistent with ischemic colitis of the cecum and ascending colon as visualized on colonoscopy from occlusion caused by the IABP. The IABP was repositioned and the patient's melena resolved.

## 2. Discussion

Intra-aortic balloon pump counterpulsation in the use of heart failure was first described by Kantrowitz in 1968 [1]. It functions primarily by augmenting diastolic function and reduction of afterload. It is generally placed via the femoral artery into the descending aorta. During diastole the balloon inflates increasing diastolic pressure [1]. During systole the balloon rapidly deflates creating a suction and thereby decreases aortic pressure and reduces afterload [1]. Frequency of placement of an IABP and its indications has been increasing since its first use and has been shown to prolong survival in critically ill cardiac patients. While the patients requiring these devices are typically critically ill and already have a degree of hypoperfusion, malpositioning of these devices has been associated with complications including further vascular hypoperfusion and bowel infarction that is often poorly tolerated by these patients.

Ischemic colitis is the most common form of intestinal ischemia and accounts for 50–60% of all cases [2]. The etiologies are numerous and can range from atherosclerotic and inflammatory processes causing occlusive disease to sepsis and heart failure causing nonocclusive hypoperfusion [2]. Regardless of the cause, patients present with acute onset abdominal pain that is cramping followed by the development of hematochezia. Other symptoms include diarrhea in 68% of cases and nausea and vomiting in 38% of cases [2]. About 3 to 9% of all cases of lower gastrointestinal bleeding can be attributed to ischemic colitis [2]. The initial diagnosis is often made based on history and clinical findings with the combination of laboratory values such as elevated lactate levels as well as imaging using CT. Confirmation is established using direct visualization via endoscopy which is the current gold standard for diagnosis [2].

In current practice, correct placement of an IABP is often confirmed using fluoroscopy or chest radiography [3]. The typical landmark used is the IABP tip just distal to the aortic knob [3]. While these can be helpful in detecting malpositioning, there continues to be a growing incidence of malpositioned IABP leading to potentially further visceral compromise and poor outcomes.

A study conducted by Yap et al. involved a literature review addressing the question if it is possible to predict the risk of ischemic bowel after cardiac surgery. The review found 7 studies that represented 68,214 patients from 7 cardiac centers from 1980 to 2011. Five of the seven papers reviewed had reported the use of an IABP was a significant risk factor [4].

A systematic review was done by Rastan et al. to evaluate the clinical significance and causes of IABP malpositioning in patients with available CT imaging [5]. 621 patients who underwent IABP placement for cardiac support between February 2007 and March 2009 were evaluated. 63 (10.1%) of the patients also underwent abdominal CT imaging. In 61 of the 63 patients evaluated, compromise of at least one visceral artery was noted including the celiac trunk (96.8%), superior mesenteric artery (87.3%), and renal arteries (66.7%). 23.8% of the patients required laparotomy for mesenteric ischemia with a mortality rate of 60.3%. Based on this study we can see that there is a significant risk to visceral perfusion with IABP placement and that this was identified by CT imaging.

Siriwardena et al. performed an evaluation of IABP malpositioning and its effects on outcomes [6]. They conducted a retrospective review on IABP in two Australasian centers and evaluated how the IABP tip position can have an effect on outcomes. 645 cases were reviewed from the 2 centers and the overall complication rates after IABP were 24.3% and 26.2%. Analysis of those cases showed the IABP to be malpositioned in 11% and severely malpositioned in 6% and unavailable in 43%.

We present a case of ischemic colitis of the cecum and ascending colon caused by malpositioning of an intra-aortic balloon pump counterpulsation device. Although bowel ischemia has been described before in the setting of IABP, the location of ischemia in the cecum and ascending colon as presented in this case is not typical. While the life-saving nature of these devices can be vital, patients should be monitored intensively for potential complications due to hindered organ perfusion including bowel ischemia. We recommend obtaining serum lactate levels in the appropriate clinical setting. We also recommend postplacement imaging using CT as opposed to chest radiograph to confirm correct placement especially in patients with increasing lactate. We believe early detection using these modalities would lead to earlier repositioning of the IABP and decrease overall complications and mortality. In addition, further studies for researching the cost effective methods of ensuring adequate placement will be needed as well as exploring methods for monitoring adequate perfusion in this already frail patient population.
